# A Novel Nanofiber Hydrogel Adhesive Based on Carboxymethyl Cellulose Modified by Adenine and Thymine

**DOI:** 10.3390/polym16071008

**Published:** 2024-04-07

**Authors:** Chong Xie, Runde Yang, Xing Wan, Haorong Li, Liangyao Ge, Xiaofeng Li, Guanglei Zhao

**Affiliations:** 1 State Key Laboratory of Pulp and Paper Engineering, School of Light Industry and Engineering, South China University of Technology, Wushan Road, Guangzhou 510641, China; 201810107072@mail.scut.edu.cn (C.X.); 202021029251@mail.scut.edu.cn (R.Y.); 202121030223@mail.scut.edu.cn (X.W.); 202220128588@mail.scut.edu.cn (H.L.); ligeliangyao@mail.scut.edu.cn (L.G.); 2 School of Food Science and Engineering, South China University of Technology, Wushan Road, Guangzhou 510641, China

**Keywords:** carboxymethyl cellulose, adenine, thymine, nanofiber gel adhesive

## Abstract

Natural polymer-based adhesive hydrogels have garnered significant interest for their outstanding strength and versatile applications, in addition to being eco-friendly. However, the adhesive capabilities of purely natural products are suboptimal, which hampers their practical use. To address this, we engineered carboxymethyl cellulose (CMC) surfaces with complementary bases, adenine (A) and thymine (T), to facilitate the self-assembly of adhesive hydrogels (CMC-AT) with a nanofiber configuration. Impressively, the shear adhesive strength reached up to 6.49 MPa with a mere 2% adhesive concentration. Building upon this innovation, we conducted a comparative analysis of the shear adhesion properties between CMC and CMC-AT hydrogel adhesives when applied to delignified and non-delignified wood chips. We examined the interplay between the adhesives and the substrate, as well as the role of mechanical interlocking in overall adhesion performance. Our findings offer a fresh perspective on the development of new biodegradable polymer hydrogel adhesives.

## 1. Introduction

Hydrogel adhesives have become ubiquitous in surface engineering, serving to bind a plethora of substrates together [1]. Synthetic polymers derived from petroleum, such as polyacrylamide [2], polyacrylic acid [3], polyethylene glycol [4], and various specially crafted hydrogels, harness interactions with substrates or employ sophisticated structural tuning techniques to secure high adhesive strengths [5]. Nonetheless, growing concerns about environmental and human health risks, coupled with the global drive towards carbon neutrality, have shifted the spotlight onto hydrogel adhesives derived from natural polymers like cellulose [6], chitosan [7], and proteins [8]. Despite the burgeoning interest, enhancing the adhesive prowess of natural hydrogels typically necessitates blending with petroleum-based polymers or engaging in complex modification procedures—factors that can curtail their broader adoption [9,10,11].

Carboxymethyl cellulose is a commercially mature chemical product. This anionic cellulose water-soluble derivative has a wide range of applications [12,13] in food [14], textiles [15,16], pharmaceutical engineering [17], biomedical engineering [18], energy storage [19], and other fields due to its unique surface properties, biological adaptability, adjustable hydrophilicity, low cost, and abundant raw material sources. Due to its abundant hydroxyl groups, CMC can serve as a hydrogen bonding site, and its anionic polyelectrolyte properties can provide long-range electrostatic interactions, providing reliable cohesion and adhesion for CMC as an adhesive [20,21,22,23]. Meanwhile, the excellent water absorption ability of CMC can maintain excess water when used as a binder [24]. So far, many researchers have devoted themselves to developing various functional CMC hydrogel adhesives. They modify the surface of CMC-based hydrogels by doping polymers, such as cellulose nanocrystalline CNC [25], chitosan [26], polyacrylamide [27], and polydopamine [28], or by using structures with an adhesive function such as dopamine to give them stronger adhesion or adhesion to special surfaces.

In the composition of the bonding strength between the adhesive and the substrate, in addition to the adhesive force contributed by the interaction, the mechanical interlocking generated by structural coupling is also especially important. Cellulose nanofibers can form structural bonds on the surface of the substrate, and their high modulus may contribute to stronger mechanical interlocking. Modulating the rigidity of the molecular chain can balance the adhesive effect and mechanical interlocking, and even form anisotropic adhesive effects based on the assembled structure. This interesting mechanism has great potential for application. Currently, CMC nanofibers are obtained by the high-pressure homogenization of carboxylate cellulose [29] or by doping PVA, as well as other electrostatic spinning processes [30]. However, their excessive cost and less environmentally friendly process hinder the large-scale preparation and application of CMC nanofiber adhesives to a certain extent. Therefore, we propose a method based on the pairing behavior of base pairs [31] and their different aggregation behaviors in water by modifying adenine and thymine on the surface of CMC and allowing them to self-assemble in water to obtain CMC nanofibers from the bottom up.

**Figure 1:**
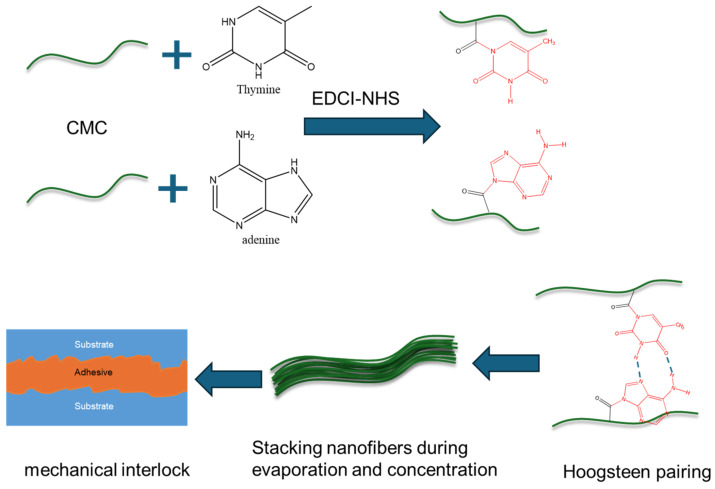
Schematic diagram of preparation process and adhesion mechanism of CMC-AT nanofiber hydrogel adhesive.

Here, we developed a strong adhesive (CMC-AT) CMC nanofiber hydrogel for the first time. The adhesive was prepared by agitating adenine A and thymine T with CMC through the EDCI-NHS catalytic system, and then slowly evaporating the solvent after mixing (Figure 1). The reaction of adenine and thymine on the surface of CMC and the aggregation structure of nanofibers were analyzed by XRD and FTIR, alongside the morphological characterization of CMC-AT by SEM. We measured the tensile strength and elastic modulus of CMC-AT and CMC and compared their shear adhesion forces on basswood chips and delignified basswood chips, respectively. Wood is composed of cellulose, hemicellulose, and lignin. There is much lignin on the surface of wood, which is composed of guaiacyl, syringyl, and p-hydroxyphenyl groups containing benzene rings [32]. After delignification treatment with acetic acid and sodium chlorite, the surface of wood is composed of cellulose and a small amount of hemicellulose [33]. However, the cellulose structure is like CMC, and many hydroxyl groups ensure similar affinity interactions. However, we modified the surface of CMC with adenine and thymine (CMC-AT), which are structurally like lignin, containing many π electrons, and have similar affinity interactions. Therefore, we constructed two different surface adhesion conditions based on the difference in structural similarity to verify the dominant role of the mechanical interlocking mechanism in the adhesion of this adhesive.

## 2. Materials and Methods

### 2.1. Experimental Materials

Sodium carboxymethyl cellulose (Na-CMC) (average molecular weight of 250,000 g mol^−1^ with a substitution degree of 1.2 [34]), 1-(3-Dimethylaminopropyl)-3-ethylcarbodiimide Hydrochloride (EDCI), N-Hydroxy succinimide (NHS), adenine (A), and thymine (T) were procured from Sigma-Aldrich (St. Louis, MO, USA).

### 2.2. Preparation of CMC-AT Nanofiber Hydrogel Adhesive

According to the report, different EDCI/NHS and buffer systems were considered [35,36,37,38,39]. It was found that when the ratio of NHS to EDCI exceeded one, there was almost no unreacted adenine and thymine. Using an MES buffer system, adenine and thymine were easily precipitated during the reaction. Using hydrochloric acid instead of PBS buffer can play the same role in dissolving adenine and thymine, but it requires a lower pH, which may be detrimental to the reaction.

In a PBS buffer (pH = 5), carboxymethyl cellulose sodium (CMC) was dissolved to achieve a concentration of 2 wt.% overnight. A predetermined quantity of adenine and NHS was then added to the CMC solution and stirred for 30 min. Subsequently, EDCI was added to the reaction solution in equimolar amounts, and the reaction was allowed to proceed for 24 h at room temperature; the molar ratio of carboxyl groups in EDC, NHS, adenine (A), thymine (T), and CMC was 10:10:4:4:10. Due to adenine’s low water solubility at room temperature, it was necessary to add it in small increments multiple times. This procedure was similarly required for the thymine group. Following this, the reaction products of both the adenine and thymine groups were mixed and stirred together at room temperature for another 24 h. The final product was then purified using PBS buffer (pH = 5) and deionized water through dialysis (MWCO: 8000–14,000 Da).

The water solubility of adenine and thymine is sensitive to pH, and they dissolve in dilute acid but hardly dissolve in neutral aqueous solutions. Therefore, if there is residual unreacted adenine and thymine in the system, directly washing or dialysis with deionized water will cause the unreacted adenine or thymine to directly precipitate as crystals or nanoparticles mixed with the adhesive. Therefore, it is necessary to ensure that unreacted adenine and thymine are removed under stable acidic conditions. In fact, this process will remove unreacted adenine and thymine as well as water-soluble EDCI and NHS. The purpose of dialysis with deionized water is to remove the PBS buffer.

During the dialysis process, the solution was monitored by ultraviolet–visible light spectroscopy to ensure the complete removal of free bases and other catalysts. After treatment with PBS buffer, during the dialysis process of deionized water, only the peak of PBS buffer was found in the UV absorption spectrum at 260 nm, indicating that other catalysts had been removed before this. We performed deionized water dialysis until the UV absorption peak of the PBS buffer no longer appeared. The mass concentration of the mixed CMC was controlled to be equivalent to 2 wt.%.

### 2.3. Characterizations

To investigate the chemical reaction between CMC and adenine and thymine, the samples were analyzed using Fourier transform infrared spectroscopy (FTIR) and attenuated total reflection (ATR) prestige-21 (Shimadzu, Kyoto, Japan). The FTIR-ATR spectrum was taken from 600 to 4000 cm^−1^. The morphology of the samples was examined using a scanning electron microscope (SEM) (SU8000, HITACHI, Ichihara, Japan). The acceleration energy of all samples was maintained at 5 kV. In addition, 600 MHz solid-state nuclear magnetic resonance was used to analyze the chemical structure of CMC and CMC-AT (BRUKER AVANCE NEO 600WB, Karlsruhe, Germany). The crystal structures of CMC and CMC-AT were observed by X-ray diffraction (XRD) spectroscopy. The XRD pattern was taken at room temperature (25 ± 3 °C) using a Rotaflex RT300 mA, Osaka, Japan, with an angle (2θ) range of 5 ≤ 2θ ≤ 60°. The mechanical properties of the prepared CMC and CMC-AT films were evaluated using a universal testing machine (UTM), the Tensilon RTC 250A, A&D Company Ltd., Osaka, Japan.

### 2.4. Shear Adhesion Test

To quantify the adhesion effect of the CMC-AT hydrogel adhesive, CMC and CMC-AT were used to glue the overlapping area (5 mm × 5 mm) of two basswood pieces (25 mm × 5 mm × 2 mm) to prepare samples. This sample does not require pressure or temperature, and only needs to be left at room temperature for 1 day to assess the shear adhesion strength. The mass concentration of CMC and CMC-AT used is 2 wt.%, with a coating amount of 150 μL. We prepared samples using delignified basswood chips as the substrate in the same way. To evaluate the upper limit of the adhesive potential of CMC-AT, we applied CMC and CMC-AT in three coats on the adhesive area of the wood chips and tested the shear adhesion strength. All shear strength tests were conducted using a universal testing machine (Sun, UTM2503, Shenzhen, China).

## 3. Results and Discussion

### 3.1. Morphological Characterization

**Figure 2:**
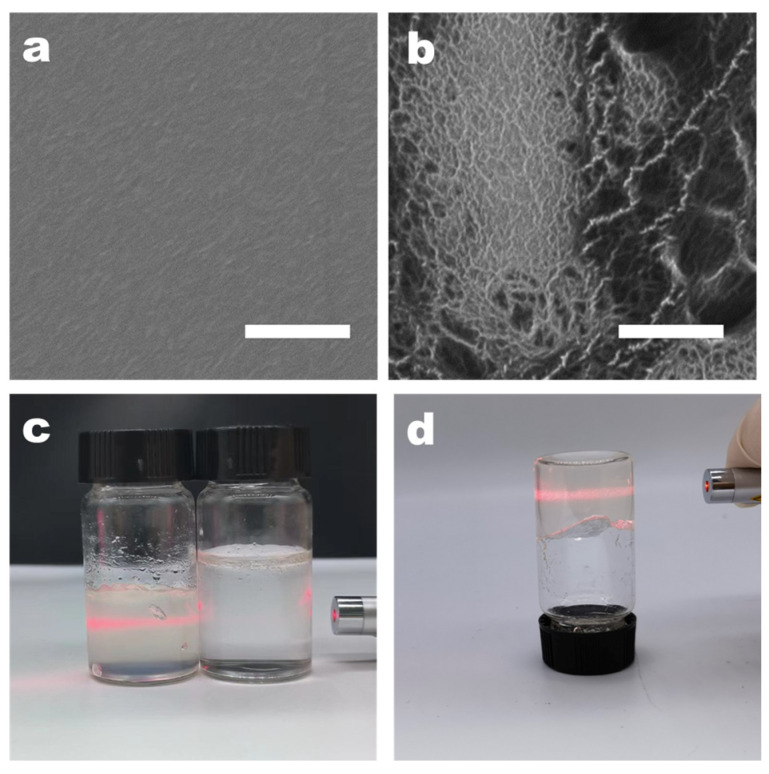
SEM image of membrane: (**a**) CMC AND (**b**) CMC-AT; the white scale bar in the figure is 1 μm. Images of aqueous solutions: (**c**) Tyndall effect of 2 wt.% CMC on the right and CMC-AT on the left; (**d**) inverted 2 wt.% CMC-AT.

The surface structure of CMC nanofibers, before and after nanofibering, was examined using high-resolution scanning electron microscopy (SEM). The SEM images revealed that the surface of the pure CMC film was smooth, lacking any typical signs of local aggregation or nanofiber characteristics (Figure 2a). This smoothness was attributed to the good water solubility of CMC, with the solution being evenly dispersed at the molecular level prior to film formation. During the stage of dialysis with deionized water, the pH gradually changes to neutral. At this time, adenine and thymine modified on the surface of CMC may induce aggregation. This part is based on the hypothesis that adenine and thymine are insoluble in neutral deionized water. We concentrated the dialyzed CMC-AT by rotary evaporation and balanced it with water to ensure a concentration of 2 wt.% with CMC, and they exhibited different properties. Different from the viscosity of CMC, CMC-AT is in a hydrogel state at this concentration, as shown in Figure 2c, d, and there is a Tyndall effect, indicating that nano fibrosis occurred at this time. As the solvents evaporated during the concentration process, the CMC aggregated evenly, resulting in a smooth and uniform film. In contrast, CMC-AT, which consisted of CMC mixed with adenine and thymine modifications, displayed distinct nanofiber structural features (Figure 2b). AN analysis through THE ImageJ software (version 1.8.0) determined that the diameters of the nanofibers ranged from 10 to 20 nm. These characteristics could be ascribed to the propensity of adenine and thymine modifications on the CMC surface to aggregate in water at room temperature, due to base pairing or π–π stacking interactions. During dialysis or as the solvent concentration increased via evaporation, this led to uneven aggregation and the subsequent formation of nanofiber structures, following a bottom-up strategy.

### 3.2. Fourier Transform Infrared Spectroscopy Analysis

**Figure 3:**
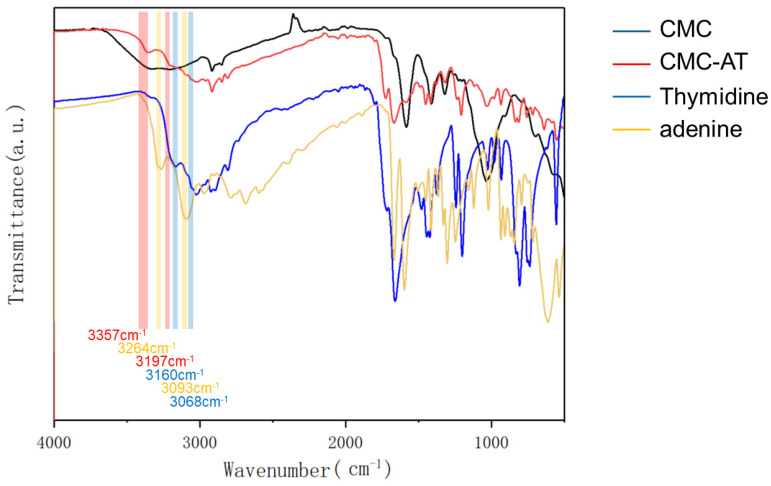
FTIR spectra of CMC, CMC-AT, thymidine, and adenine.

Adenine and thymine reacted with carboxymethyl cellulose under the catalysis of EDCI-NHS to form a tertiary amide structure. The peak at 1670 cm^−1^ and the weak peak at 1546 cm^−1^ corresponded to the stretching vibration peak of the tertiary amide C=O group and the characteristic peak of the amide II band, respectively. The FTIR analysis also provided some indication of the aggregation form of the adenine and thymine groups. In fact, there are two typical forms of base complementarity: Watson–Crick pairing [40,41] and Hoogsteen pairing [42]. Compared to Watson–Crick pairing, the A base rotates by an angle and uses other atoms to form complementary pairing with the T base. In Watson–Crick pairing, the N1 and N on the C6 of the A base form two hydrogen bonds with the O on the N3 and C4 of the T base, respectively. In Hoogsteen pairing, the N7 and N on the C6 of the A base form two hydrogen bonds with the O on the N3 and C4 of the T base. Meanwhile, G and C bases can also form hydrogen bonds with each other at other angles, but only two hydrogen bonds are formed instead of three. Some studies showed that in the liquid phase, Hoogsteen pairing is dominant, exhibiting typical characteristics of infrared absorption peaks. As demonstrated in Figure 3, the N-H stretching vibration peaks of the individual adenine and thymine aggregates were located at 3264 cm^−1^ and 3093 cm^−1^, respectively. For adenine and thymine paired in Hoogsteen form, these shifted to 3160 cm^−1^ and 3068 cm^−1^. Moreover, once adenine and thymine were paired in Hoogsteen form, the stretching vibration peaks appeared at 3357 cm^−1^ and 3197 cm^−1^. Notably, the 3357 cm^−1^ peak after pairing slightly blue-shifted from the literature value of 3395 cm^−1^ [42]. This shift was due to the substitution of hydrogen on the secondary amine on the purine ring by a carbonyl group, which increased the conjugated structure.

### 3.3. Solid-State Nuclear Magnetic Resonance Analysis

**Figure 4:**
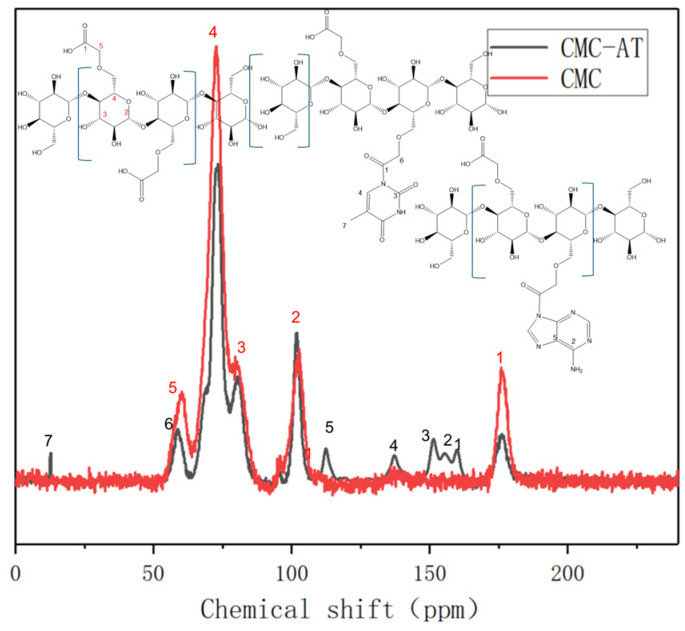
Solid-state nuclear magnetic resonance carbon spectra of CMC and CMC-AT.

Although CMC can be dissolved in water, CMC-AT modified with adenine and thymine and nanostructured is insoluble in common solvents such as water, dilute acid, DMSO, and acetone. Therefore, its solid-state carbon magnetic resonance spectra were characterized and analyzed. The typical characteristic peak of the carboxyl carbon chemical shift of CMC is 176 ppm, and the peak intensity of this chemical shift significantly decreases after the reaction (Figure 4, red peak No. 1). This proves that the carboxyl group underwent a chemical reaction. The chemical shift of the ethyl carbon attached to it moved from 60 ppm to 58 ppm, indicating a decrease in the degree of shielding of the electron-withdrawing group. This may be due to the reaction between the carboxyl group and the secondary amines on the adenine ring of the conjugated structure, forming a larger conjugated structure and increasing resonance. This not only reduced the deshelling of the carbonyl carbon to move to a high field of 161 ppm, but also caused the ethyl carbon attached to it to move 2 ppm to a high field. The carboxyl group on CMC reacts with the secondary amine on adenine to form a tertiary amide structure. The carboxyl group on CMC reacts with the secondary amine on thymine between the methyl and carbonyl groups to form a dendritic amide structure. The chemical shifts corresponding to the five carbons on adenine are 155.3, 152.37, 151.3, 139.29, and 117.61 ppm, respectively [43]. The chemical shifts corresponding to the five carbons on thymine are 163.91, 151.34, 141.93, 107.19, and 12.51 ppm, respectively [44]. Due to the small number of hydrogen atoms on the ring, the signal is not as strong as that of the carbon atoms on the CMC chain. After the reaction, the formation of amide groups extends the conjugated structure, causing a slight shift to higher fields. The chemical shift of the amide carbon is close to that of one of the carbon groups on the thymine ring. These groups are close and inevitably form continuous peaks due to the resolution of solid-state nuclear magnetic resonance.

### 3.4. X-ray Diffraction Analysis

**Figure 5:**
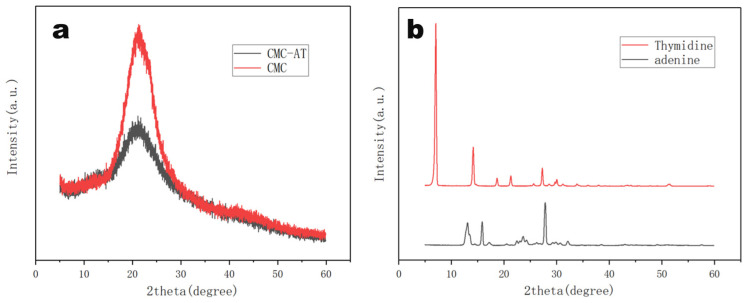
X-ray diffraction (XRD) spectra of (**a**) CMC, CMC-AT, (**b**)thymidine, and adenine.

Unreacted adenine and thymine were easy to crystallize in water at room temperature. The X-ray diffraction analysis of CMC and CMC-AT reflected their aggregation statuses. As illustrated in Figure 5a, both CMC-AT and CMC exhibited typical CMC characteristic peaks at 22°. However, compared to pure CMC, the crystallinity of CMC-AT was significantly reduced, and no crystallization peaks of adenine and thymine appeared (Figure 5b). According to the peak area integration method, the crystallinity of CMC is 64.3%, while that of CMC-AT is 43.1%. Based on this result, it could be considered that the surface modification of CMC with adenine and thymine inhibited the direct contact of some CMC molecules, thereby reducing the crystallinity. Combined with the analysis results of FTIR, the pairing of adenine and thymine might have completed this part of the aggregation.

### 3.5. Tensile Strength Test

**Figure 6:**
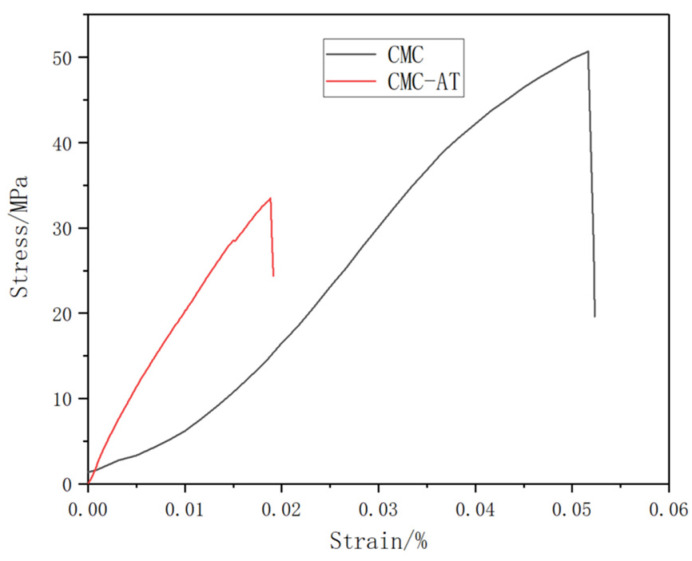
Stress–strain curves of CMC and CMC-AT.

The mass concentration of 2 wt.% CMC and CMC-AT were directly dried at 60 °C for 24 h to form a film, and then the film was placed in a vacuum drying oven at 50 °C for 24 h to completely remove free water. The test results showed that (Figure 6) the tensile strength of the CMC film was 50.68 MPa, while the elastic modulus was 1.41 GPa. The tensile strength of CMC-AT was 33.49 MPa, and the elastic modulus was 2.51 GPa. CMC-AT had lower tensile strength than CMC, but a significantly higher modulus, due to its less compact structure. As an adhesive, mechanical interlocking is a vital component of adhesive strength, and a higher modulus leads to less deformation, thus enhancing this effect.

### 3.6. Adhesion Condition of Wood Surface

**Figure 7:**
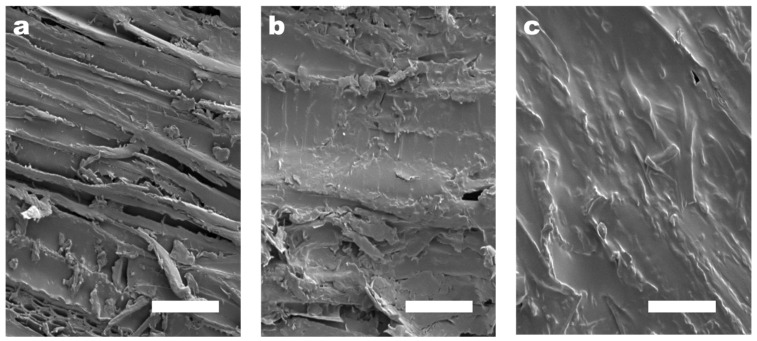
SEM image of the surface of the basswood chips with different coating amounts of CMC-AT: (**a**) 0, (**b**) 120 g/m^2^, (**c**) 360 g/m^2^; the white scale bar in the figure is 50 μm.

The surfaces of porous and loose basswood pieces were polished smooth with one-thousand-grit sandpaper to avoid experimental errors caused by the randomness of natural raw materials. The concentration of the CMC-AT hydrogel adhesive used in bonding was limited to 2 wt.%, because the saturation concentration of the CMC, purchased with a degree of substitution (DS) of 1.2, in deionized water was lower than 2.5 wt.%. To ensure that both CMC and CMC-AT had the same concentration during adhesion testing, a consistent 2 wt.% concentration was employed as the adhesive concentration. On the other hand, drying the hydrogel to achieve a higher adhesive concentration was feasible for application purposes. However, in the experiment, such a procedure would lead to the uneven distribution of surface and internal moisture within the gel, thereby causing experimental errors. As a result, the amount of CMC-AT adhesive that could be applied in a single instance on a limited surface area was capped due to its low concentration. If more than 150 microliters of the adhesive was applied to a 5 mm × 5 mm surface, equivalent to 120 g/m^2^, it would overflow. Hence, the quantity of adhesive used per coat was set at 150 microliters, and the surface morphology post-drying is depicted in Figure 7b. The CMC-AT hydrogel thoroughly coated the surface of the basswood, albeit with a very thin layer. In comparison to the uncoated blank surface shown in Figure 7a, the rough geometric morphology was significantly preserved. This could potentially lead to a smaller contact area during bonding, thus potentially affecting the bond strength. To achieve a greater adhesive coverage, multiple applications were required; that is, after drying for one hour, the adhesive on the surface was concentrated and then re-applied. After three coatings, the basswood surface was completely covered, as shown in Figure 7c, allowing the upper limit of the shear adhesion strength of the CMC-AT nanofiber hydrogel adhesive to be assessed at this level of application.

### 3.7. Shear Adhesion Strength Test

**Figure 8:**
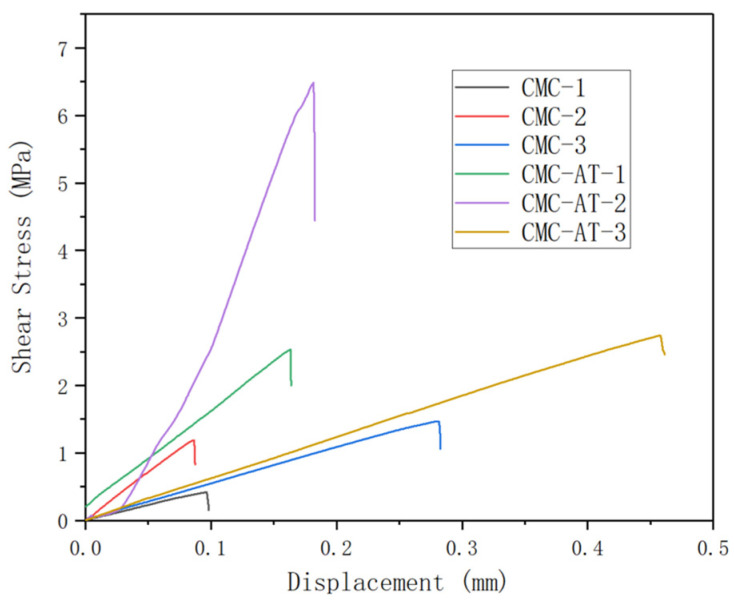
Shear adhesion strength curve.

The tensile strength corresponding to the sample numbers (Table 1) is depicted in Figure 8. The shear adhesion strength of CMC-AT bonded to basswood chips with a coating amount of 360 g/m^2^ reached 6.49 MPa. The adhesive surface was entirely coated by CMC-AT, a result of multiple applications of coatings and concentrations. The shear adhesion strength of the CMC-AT adhesive to basswood chips with a single coating of 120 g/m^2^ was measured at 2.53 MPa, with less contact observed between the adhesive surfaces. In identical conditions, CMC exhibited tensile strengths of 0.42 MPa and 1.19 MPa, respectively. The high affinity between the adenine and thymine groups in CMC-AT and lignin, as well as the higher elastic modulus, help to achieve more stable mechanical interlocking, resulting in ultra-high shear adhesion strength to wood chips. The shear bonding strength of CMC-AT on the surface of delignified lime wood chips reached 2.74 MPa, while the shear bonding strength of CMC was only 1.47 MPa. Delignified wood chips are composed of cellulose and hemicellulose components.

There are indeed limitations in the use of adhesives on different adhesive substrates. we tried to use this dose of adhesive for bonding. However, after delignification, the basswood chips composed only of cellulose and hemicellulose became overly sensitive to moisture. After applying only 2 wt.% adhesive, curling deformation inevitably occurred. In order to ensure the accuracy of the test, we tried a feasible single maximum dose of 120 g/cm^2^ to ensure that the two bonded wood chips would have the same curvature and bond together. However, for the basswood chips that were not delignified, because there was no bending problem, it was possible to repeatedly apply a maximum dose of 360 g/cm^2^ to one of the chips before performing bonding.

Compared to CMC-AT, it is obvious that CMC is easier to form hydrogen bonds with without barriers. However, for the bonding of delignified wood chips, the shear bonding strength of CMC-AT is 2.74 MPa, while the shear bonding strength of CMC is only 1.47 MPa. This clearly demonstrates the key role of mechanical interlocking in the bonding mechanism within the bonding strength. CMC-AT nanofiber hydrogel adhesive forms a stable mechanical lock due to the affinity between the matrix and lignin, the high modulus of nanofibers, and the complexity of the surface structure. This synergistic effect leads to excellent bonding strength of the Tilia tree slices. The maximum shear adhesion strength of CMC-AT to basswood is 6.49 MPa, which is obviously higher than 3.14 MPa of a modified cellulose nanorod hydrogel adhesive reported previously [45]. Compared to several recent adhesives based on natural products, it also has obvious strength advantages [46,47,48,49]. This may be related to the formation mechanism of CMC-AT nanofibers using a bottom-up strategy. During the bonding process, with the evaporation of the solvent, CMC-AT concentrates and self assembles in a directional manner, forming an extraordinarily strong mechanical interlock by forming a nanofiber microstructure in situ on the bonded surface.

## 4. Conclusions

Through the base modification and subsequent self-assembly of the CMC surface, a nanofiber hydrogel adhesive with elevated shear adhesion strength was successfully developed. The raw materials for the CMC-AT nanofiber adhesive were only CMC, water, adenine, and thymine; the adhesive boasted attributes such as biodegradability, exemplary biological adaptability, low cost, and sustainability. Remarkably, without necessitating extra pressure or temperature interventions, the adhesive achieved a maximal bond strength of 6.49 MPa to wood surfaces. This notable performance was attributable not only to the robust intermolecular interactions between the bases featuring aromatic ring structures and lignin, which shares similar aromatic ring configurations, but also to the very stable mechanical interlocking established by the nanofiber structure. This premise was further corroborated through experiments utilizing delignified basswood chips. The research outlined in this paper not only introduces a novel bottom-up approach to synthesize the CMC nanofiber structure but also delivers fresh insight into augmenting the shear adhesion strength of natural polymer hydrogels.

## Figures and Tables

**Table 1:** Amount of adhesive substrate and dosage corresponding to adhesive sample names.

Sample Names	Adhesive Substrate	Adhesive Dosage
CMC-1	basswood	120 g/m^2^
CMC-2	basswood	360 g/m^2^
CMC-3	delignified basswood	120 g/m^2^
CMC-AT-1	basswood	120 g/m^2^
CMC-AT-2	basswood	360 g/m^2^
CMC-AT-3	delignified basswood	120 g/m^2^

## Data Availability

The raw data supporting the conclusions of this article will be made available by the authors on request.
